# Arylmethylamino steroid compound 1o interferes with *Plasmodium falciparum*’*s* hemoglobin metabolism

**DOI:** 10.1128/aac.00332-25

**Published:** 2025-06-23

**Authors:** Eric Springer, Kim C. Heimsch, Niklas Sidiropoulos, Jacomina Krijnse Locker, Jude M. Przyborski

**Affiliations:** 1 Biochemistry and Molecular Biology, Interdisciplinary Research Center, Justus Liebig University 715641, Giessen, Germany; 2 Justus Liebig University, Giessen, Germany; The Children's Hospital of Philadelphia, Philadelphia, Pennsylvania, USA

**Keywords:** ATeam1.03YEMK, ATP, sfpHluorin, arylmethylamino steroids, hemoglobin, heme, malaria, *Plasmodium*

## Abstract

The anti-parasitic compound arylmethylamino steroid 1o (1o) is a promising drug candidate with low nanomolar activity against the malaria parasite *Plasmodium falciparum*, but with a so far unknown mode of action. To address this, we applied previously developed live-cell ATP and pH assays to measure effects upon exposure of parasites to 1o. Furthermore, we analyzed the parasites’ heme species distribution, the ultrastructural morphology, food vacuole (FV) appearance, and lifecycle development of different parasite stages. We found that 1o increases cytosolic [ATP] level and causes a slight drop in pH, similar to the effects of arylamino alcohols such as mefloquine. The compound also prevents chloroquine (CQ)-mediated proteolysis and limits cytosol acidification within the range of its EC_50_. Additionally, 1o prevents CQ-mediated heme and hemoglobin accumulation, and preserves ultrastructural FV integrity. Furthermore, we can demonstrate that 1o blocks the development of ring and early trophozoite stages, while late trophozoite stages were unaffected. These findings suggest that the mechanism underlying the killing activity of 1o may be the interference of a pathway within or upstream of hemoglobin digestion, particularly during the highly metabolically active earlier parasite stages. Our data open a new perspective on the compound’s mode of action, information critically needed for target identification and further drug development.

## INTRODUCTION

Malaria remains one of the greatest public health burdens for humankind, with 263 million estimated cases worldwide in 2023. Of these, around 597,000 people died, the majority of victims being children under the age of five. Among *Plasmodium* species, *Plasmodium falciparum* causes most infections and leads to the deadliest form of the disease (1). Rising resistance against antimalarials is of increasing concern, and therefore, basic research on the development of new drugs is critical for global health. Known anti-malarial drugs appear to kill the parasite by inhibition of a number of different pathways, although for several well-known drugs, the exact mode of action (MoA) is unclear or still under discussion. An understanding of the MoA of these drugs can lead to an insight into how resistances generate and spreads across populations and allow optimization of existing and future compounds to avoid the development of resistance.

The arylmethylamino steroid compound 1o (1o) is a promising drug candidate and antiplasmodial compound. It shows low nanomolar activity against chloroquine (CQ) sensitive and resistant strains, drastic cure rates in a *Plasmodium berghei* animal model (2), and demonstrates a good toxicity profile in mammalian cells and mice (2, 3). Although it has been proposed that the compound exerts its effects by covalent protein crosslinking (2), experimental evidence for a specific mechanism of action is lacking.

We used various MoA analysis techniques to advance a deeper understanding of the effects of compound 1o. We have previously used genetically-encoded biosensors to measure pH and [ATP] changes in response to various antimalarial drugs, including 4-aminoquinolines and arylamino alcohols. We observed distinct sensor-response patterns, depending on the class of compounds used (4). Here, we applied the same techniques to monitor responses to 1o. We detected increased [ATP], as well as a slight effect on pH in trophozoites upon exposure to 1o. These effects are similar to those we previously observed using established arylamino alcohol-based antimalarials, such as mefloquine (MQ). Though the exact parasite-killing mechanism of this class of compounds remains poorly understood, they are thought to exert specific effects on the parasite’s heme species distribution, cause inhibition of endocytosis, and to interfere with certain modes of action of the long-established antimalarial CQ (5–10).

Based on our initial results with the biosensors, we sought to elucidate if 1o treatment demonstrates further similarities to the arylamino alcohol class of antimalarials, including interference in hemoglobin (Hb) metabolism and interaction with CQ-induced phenotypes. These experiments reveal that 1o prevents CQ-mediated proteolysis and limits cytosol acidification within the range of its EC_50_ at 72 h (^72h^EC_50_). Additionally, it prevents CQ-mediated heme and Hb accumulation, while it preserves ultrastructural food vacuole (FV) integrity. Furthermore, we can demonstrate that 1o prevents lifecycle progression of ring and early trophozoite stages, while it hardly affects the development of late trophozoite stages. These findings suggest that the parasite-killing effect of compound 1o could be interfering with a pathway within or upstream of Hb digestion and relevant for trophozoite development.

## RESULTS

### 1o affects [ATP], pH, and parasite growth

**Fig 1:**
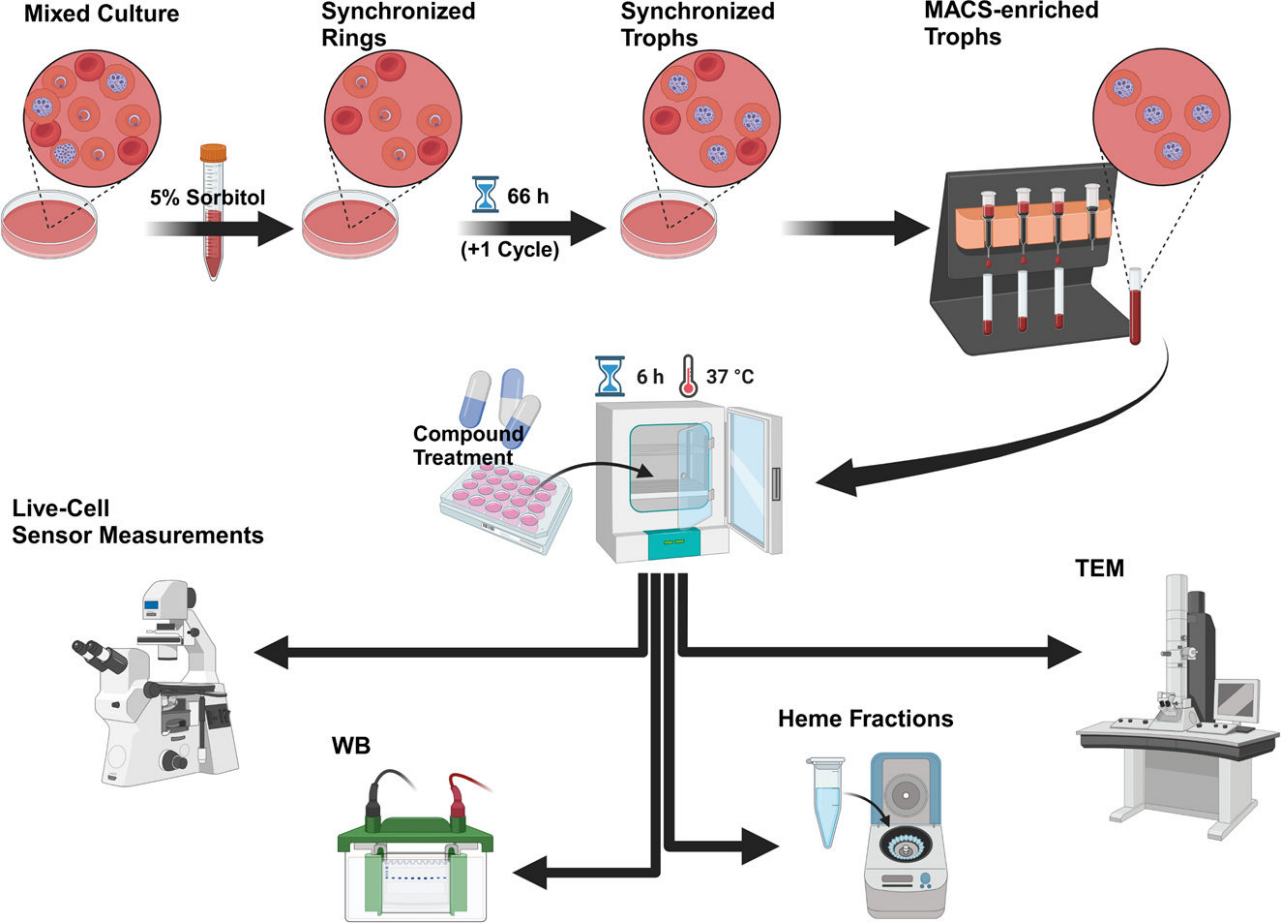
Workflow of trophozoite experiments. All parasites were synchronized for ring stages (Rings) via sorbitol for at least three consecutive cycles and were allowed to complete their last cycle prior to sample preparation. Trophozoites (Trophs) were enriched using magnetic activated cell sorting (MACS), treated with compounds or vehicle, and incubated under standard conditions prior to live-cell measurements, western blot (WB), heme fractionation, and transmission electron microscopy (TEM). Created in BioRender.

To monitor changes in [ATP] upon 1o (Fig. S1) treatment, we applied our previously established techniques (4). To ensure comparability to our previously published results, we used an identical streamlined purification and incubation design for all experiments (Fig. 1). We started our time series with mid-trophozoite stage parasites, as these stages are highly metabolically active and are also easier to image than younger stages. We were interested in compound treatments with up to 100-fold of the ^72h^EC_50_. To this end, we determined a ^72h^EC_50_ of 4.6 ± 0.1 nM for compound 1o using the NF54*attB*^[ATeam1.03YEMK]^ sensor cell line (Fig. S2A) and used this as the basis for all further experiments. Using recombinant ATeam1.03YEMK and sfpHluorin sensor protein, we could not detect significant interference within our desired concentration range, due to direct drug-sensor interactions (Fig. S2B and C).

**Fig 2:**
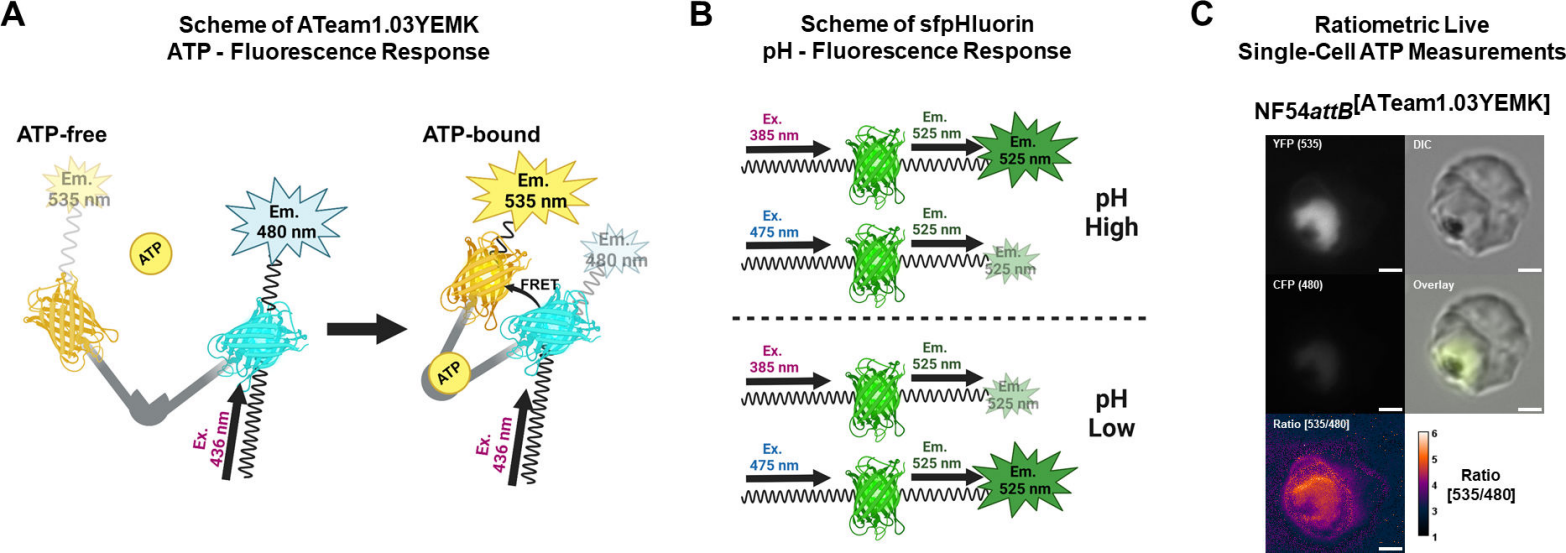
ATP, pH, and size determinations using genetically-encoded parasite lines. Sensor sequences were stably integrated into the *Plasmodium falciparum* NF54*attB* strain. (**A**) ATP measurements via ATeam1.03YEMK are based on changes in intramolecular FRET upon ATP binding, which allows ratiometric measurements of ATP levels. (**B**) The GFP-based sfpHlourin pH sensor changes its excitation properties upon pH changes, which is the basis for ratiometric measurements of the pH level. (**C**) The NF54*attB*^[ATeam1.03YEMK]^ ATP sensor cell line exemplarily demonstrates ratiometric live-cell measurements via fluorescence microscopy based on the signal intensity ratio of different fluorescence channels. The mean signal intensity after background subtraction is used for ratio calculation, while the fluorescence area is used as a proxy for the parasite size. Created in BioRender.

**Fig 3:**
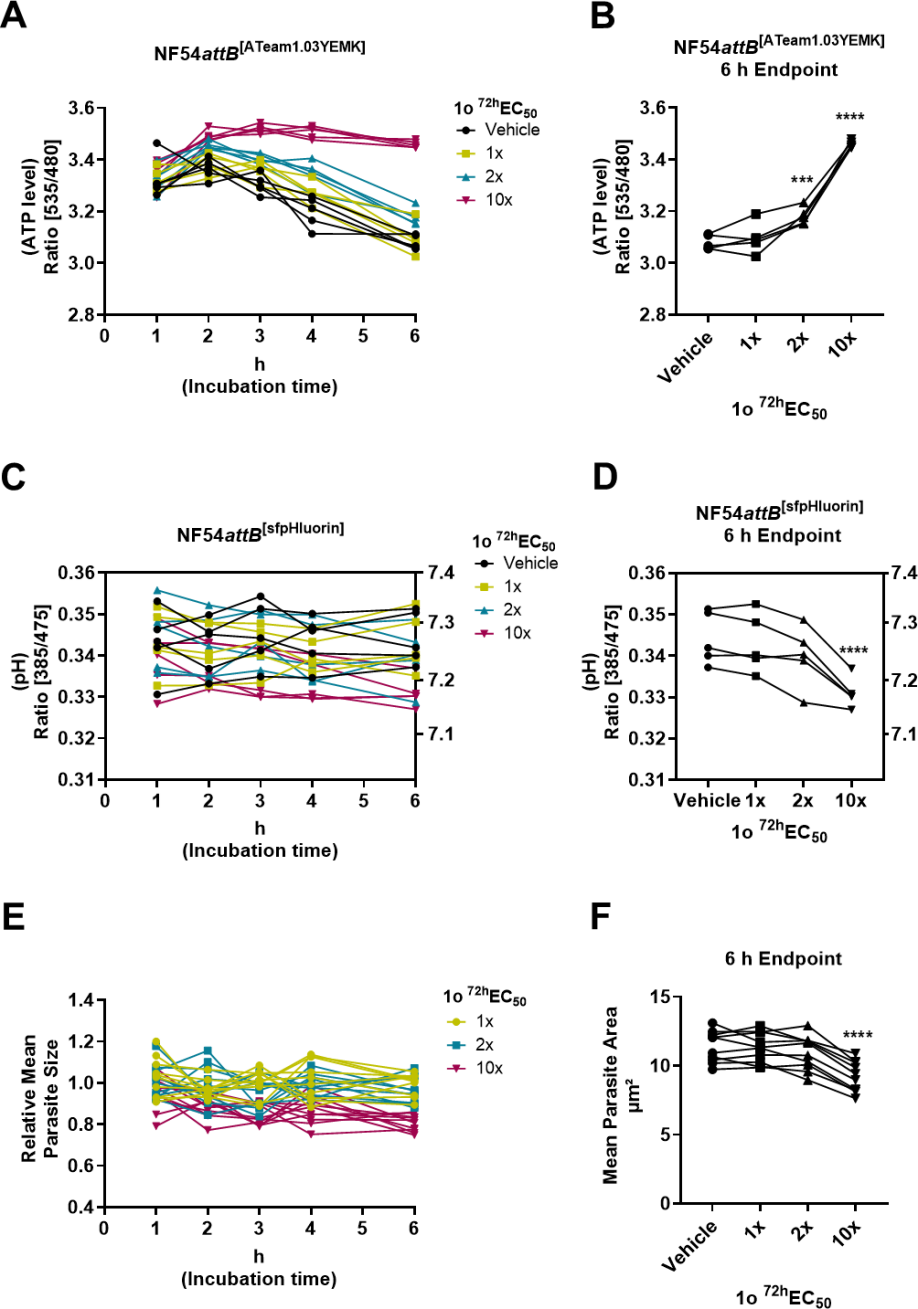
Compound 1o causes elevated ATP levels, a slight pH drop, and reduced parasite size in the cytosol of trophozoite stage parasites. *Plasmodium falciparum* sensor cell lines were used to measure ATP and pH levels of MACS-enriched trophozoite-infected red blood cells (iRBCs) via fluorescence microscopy 1, 2, 3, 4, and 6 h after incubation with multiples of the parasite-killing ^72h^EC_50_ or vehicle control. (**A and B**) ATP levels were determined using the NF54*attB*^[ATeam1.03YEMK]^ ATP sensor cell line via excitation at 430 nm and emission sensing at 535 and 480 nm for the YFP and CFP channels, respectively. The background-corrected YFP–CFP ratio is equivalent to the ATP level. After 6 h, the ATP level of 2x (*P* = 0.0004) and 10x (*P* < 0.0001) ^72h^EC_50_-treated parasites were elevated compared to vehicle-treated controls. (**C and D**) pH levels were determined using the NF54*attB*^[sfpHluorin]^ pH sensor cell line via excitation at 385 or 475 nm and emission sensing at 525 nm. Background-corrected emission ratio after 385 and 475 nm excitation is equivalent to the pH level. pH scale (right y-axis) was gained using nigericin *in cellulo* calibration. After 6 h, the mean pH (95% CI) of 10x (*P* < 0.0001) ^72h^EC_50_-treated parasites was reduced to 7.18 (7.13–7.22) compared to vehicle-treated control parasites, pH 7.27 (7.22–7.32). (**E and F**) Cytosolic fluorescence of the sensor cell lines was used as a proxy for the parasite size. The relative mean parasite size was normalized using the concurrent vehicle-treated control in each microscopic dish. (**A–D**) *n* = 5 independent experiments. Each measurement is derived from the mean of 200 parasites. Statistical analysis after 6 h was conducted via two-way analysis of variance (ANOVA) and Dunnett’s multiple comparisons test. Asterisks indicate significance level (***P < 0.001; ****P < 0.0001).

The ATeam1.03YEMK ATP sensor (11) is based on intramolecular Förster resonance energy transfer (FRET [Fig. 2A]), and its emission ratio can be used as a measure of [ATP] (4). The sfpHluorin sensor (12) changes its excitation properties upon pH changes (Fig. 2B). Additionally, we used the fluorescence of the cytosolic sensor as a proxy for parasite size (Fig. 2C). We could detect significantly elevated cytosolic [ATP] in trophozoite stage parasites at the 6 h endpoint upon treatment with 2x and 10x ^72h^EC_50_ 1o (Fig. 3A and B). We could detect a slight drop in mean cytosolic pH (95% confidence interval) from 7.27 to 7.18 (7.13–7.22) after 6 h 10x ^72h^EC_50_ 1o incubation (Fig. 3C and D). Importantly, this is still within the pH range that allows reliable [ATP] measurements (4). Analysis of images allowed us to detect significantly lower mean parasite size after 10x ^72h^EC_50_ 1o incubation compared to the control (Fig. 3E and F).

An increase in cytosolic [ATP], a drop in pH, and a reduced parasite size upon 1o treatment is a similar phenotypic pattern to that observed when treating parasites with the arylamino alcohols MQ and lumefantrine (LUM [4]). For this reason, we measured further effects known to be induced by MQ, including inhibition of CQ-mediated phenotypes, upon 1o treatment.

### 1o prevents CQ-induced sensor phenotypes around its parasite-killing ^72h^EC_50_

We previously showed that CQ causes degradation of the FRET-based ATP sensor ATeam1.03YEMK, causing distinct fluorescence and western blot (WB) signal attenuations, as well as cytosol acidification, which can be prevented by the arylamino alcohol MQ but not by the 4-aminoquinoline amodiaquine (AQ [4]). We attribute these effects to CQ-induced heme accumulation that is potentially lysing the parasite’s FV and thereby causing proteolytic degradation in the cytosol (13–17). We wished to study whether these effects, at or around the ^72^EC_50_, could be responsible for the parasite-killing activity. Thus, we determined the dose-response of 1o for correlation to its parasite-killing activity. To analyze a possible interference with Hb metabolism in more detail, we investigated effects on heme species distribution, analysis of FV integrity by transmission electron microscopy (TEM), as well as treatment of different parasite developmental stages.

**Fig 4:**
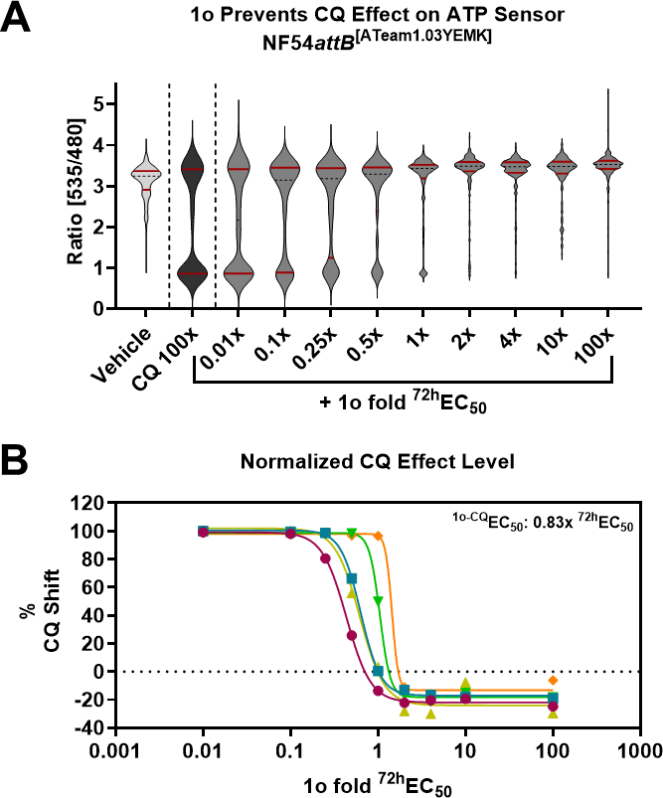
1o prevents CQ-mediated ATeam1.03YEMK ratio drop. (**A**) 100x ^72h^EC_50_ CQ causes a distinct bimodal drop of the NF54*attB*^[ATeam1.03YEMK]^ single-cell ratio distribution (black) compared to vehicle-treated control parasites (light gray) after excitation at 430 nm and emission sensing at 535 and 475 nm in MACS-enriched trophozoite iRBCs within the fluorescence microscopy setup. Co-incubation with increasing concentrations of 1o (0.01x – 100x ^72h^EC_50_; light gray) prevents the appearance of a CQ-induced lower distribution mode in a dose-dependent manner. The solid red lines indicate the 25th and 75th percentiles. (**B**) Normalization of 1o’s effects on the 25th percentile and plotting on a sigmoidal 4-factoral dose-response curve allows the calculation of a half-maximal effective dose of 1o on the CQ phenotype (^1o-CQ^EC_50_). *N* = 5 independent experiments are shown. The mean (±SD) ^1o-CQ^EC_50_ is equivalent to 0.83x (±0.34) of the parasite-killing ^72h^EC_50_. Different colored points and fit lines indicate independent replicates.

**Fig 5:**
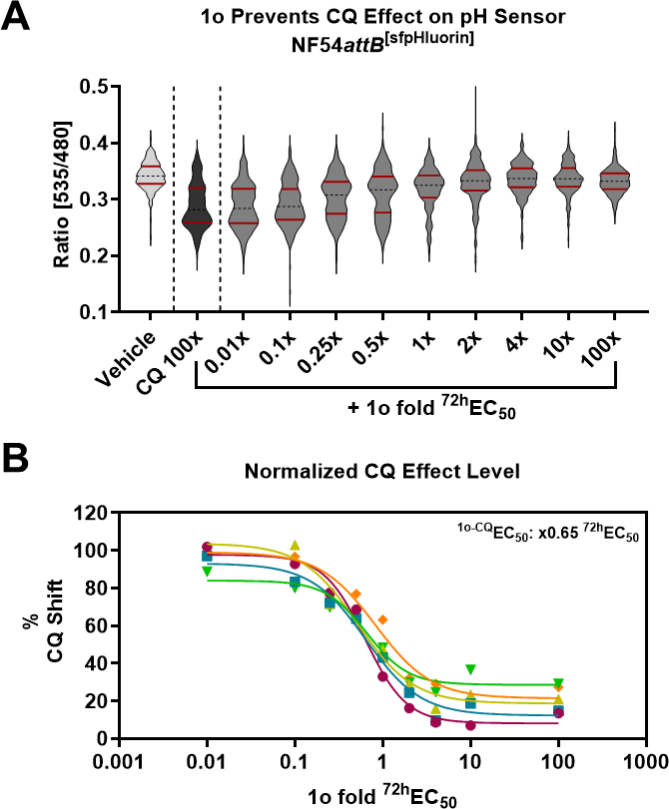
1o prevents CQ-mediated sfpHluorin ratio drop. (**A**) 100x ^72h^EC_50_ CQ causes a distinct bimodal shift of the NF54*attB*^[sfpHluorin]^ single-cell ratio distribution (black) compared to vehicle-treated control parasites (light gray) after excitation at 385 and 475 nm with emission sensing at 525 nm in MACS-enriched trophozoite iRBCs within the fluorescence microscopy setup. Co-incubation with increasing concentrations of 1o (0.01x – 100x ^72h^EC_50_; light gray) prevents the appearance of a CQ-induced lower distribution mode in a dose-dependent manner. The solid red lines indicate the 25th and 75th percentiles. (**B**) Normalization of 1o’s effects on the 25th percentile allows the calculation of a half-maximal effective dose of 1o on the CQ phenotype (^1o-CQ^EC_50_). *n* = 5 independent experiments are shown. The mean (±SD) ^1o-CQ^EC_50_ is equivalent to 0.65x (±0.10) of the parasite-killing ^72h^EC_50_. Different colored points and fit lines indicate independent replicates.

We recently showed that treatment of parasites with 690 nM CQ (100x EC_50_) causes a distinct bimodal single-cell ATP and pH level distribution, and this effect can be prevented through co-incubation with MQ (4). Co-incubation of trophozoite stage parasites of the ATP sensor cell line with 100x ^72h^EC_50_ CQ and increasing concentrations of 1o (0.01x – 100x ^72h^EC_50_) reveals that 1o prevents this CQ phenotype, in an apparent dose-dependent manner (Fig. 4A). The bimodal data distribution of the CQ-treated parasites shows an apparent correlation with the 25th and 75th percentile markers (Fig. 4A). In particular, the 25th percentile marker was representative of the CQ effect (in comparison to vehicle control). To enable further data analysis, we thus used this 25th percentile marker as a numerical surrogate for the CQ effect. We then used this marker for quantification of the CQ effect level. We normalized the 25th percentile ratio of the 1o co-incubations (CQ set to 100%; vehicle set to 0%) for dose-response analysis (Fig. 4B). We calculated the half-maximal effective concentration for this 1o-CQ interaction (^1o-CQ^EC_50_) and found that 1o prevents the CQ-mediated phenotype with 0.83x ± 0.36 (mean ± SD) of its parasite-killing ^72h^EC_50_ (Fig. 4B). As recently shown, 100x ^72h^EC_50_ CQ also causes a ratio distribution shift in trophozoites of the pH sensor cell line NF54*attB*^[sfpHluorin]^ responsive to MQ (4). Using the identical experimental conditions of the previously described experiment for NF54*attB*^[sfpHluorin]^, we found that 1o also prevents the CQ-characteristic sfpHluorin ratio distribution in a dose-dependent manner (Fig. 5A). Again, we used the 25th percentile as a marker for the CQ phenotype and determined the ^1o-CQ^EC_50_ to equal 0.65x ± 0.10 (mean ± SD) of the parasite-killing ^72h^EC_50_ (Fig. 5B).

### 1o prevents CQ-induced sensor degradation

**Fig 6:**
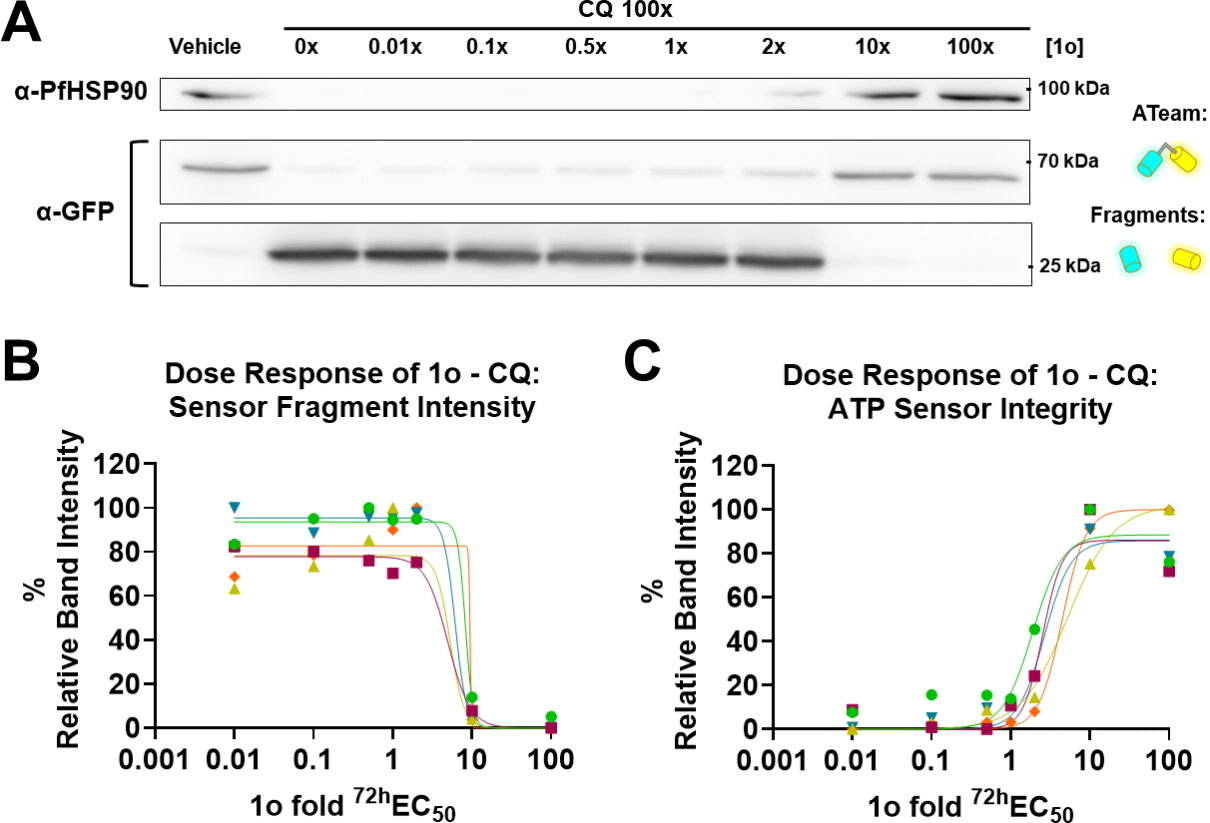
1o prevents CQ-mediated ATeam1.03YEMK sensor degradation. (**A**) WB of MACS-enriched NF54*attB*^[ATeam1.03YEMK]^ trophozoite iRBCs treated with 100x ^72h^EC_50_ CQ co-incubated with increasing concentrations of 1o (0.01x – 100x ^72h^EC_50_) for 6 h. CQ causes the disappearance of the housekeeping control (PfHSP90; 90 kDa) and the GFP-based ATeam1.03YEMK (68 kDa) signal, concomitant with increased signal intensity around the size of GFP-based monomers (27 kDa; Fragments). (**B and C**) Normalization of WB to vehicle control and CQ alone signals shows a dose dependency. Band intensities were normalized to Ponceau S total protein stain. *n* = 5 independent experiments with 2.5 × 10^6^ parasites per group are shown. Different colored points and fit lines indicate independent replicates.

In our previous study, we found that the CQ-mediated bimodal drop of the ATeam1.03YEMK ratio is accompanied by degradation of the ATeam sensor and the PfHSP90 housekeeping control protein on WB, which could be prevented by MQ (4). To investigate 1o’s capabilities to prevent such degradation in red blood cells (RBCs) infected with trophozoite stage NF54*attB*^[ATeam1.03YEMK]^ parasites, we used the identical experimental setup of the two previously described experiments and carried out WB analysis. Using an anti-GFP antibody that recognizes ATeam, we could detect a signal just below 70 kDa, representing intact ATeam1.03YEMK sensor (68 kDa), as well as a faint signal intensity around 25 kDa, correlating to protease-resistant GFP-based degradation products in the vehicle control parasites (Fig. 6A). CQ treatment causes the disappearance of the ATeam signal, while the signal from GFP degradation products drastically increases. Co-incubation with increasing 1o concentrations prevented ATeam degradation and fragment appearance. We could detect a similar phenomenon while analyzing PfHSP90. CQ treatment caused the disappearance of the PfHSP90 signal, while increasing 1o concentrations could prevent this effect. Normalization of the sensor fragment (Fig. 6B) and the ATeam sensor intensity (Fig. 6C) demonstrates a dose-dependent relationship of the described 1o-CQ interaction.

### 1o alters the parasites’ heme species distribution

**Fig 7:**
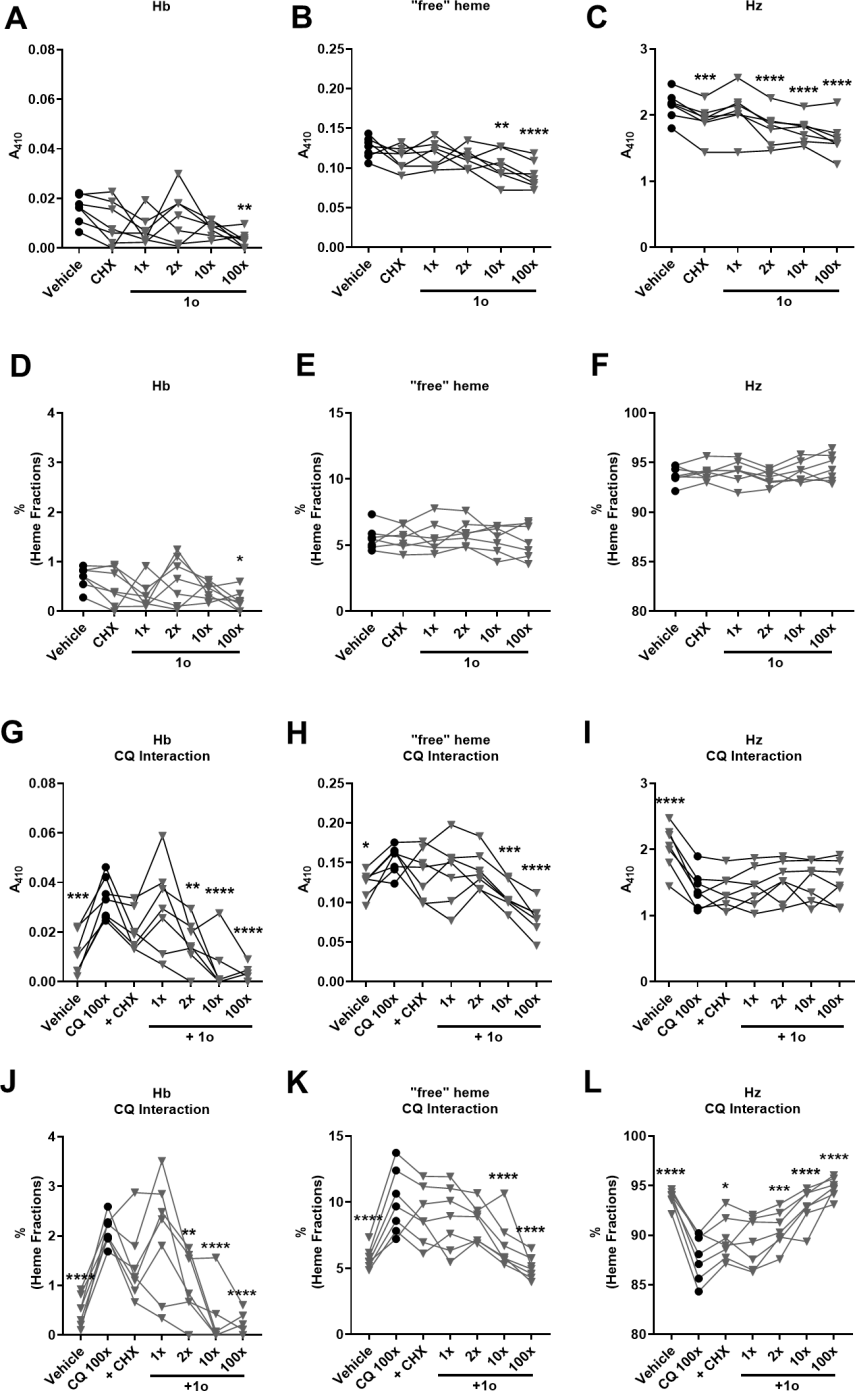
1o depresses Hb metabolism, lowers Hb fraction, and prevents CQ-mediated effects on Hb and “free” heme species. Fractionated analysis of heme species (Hb: hemoglobin; “free” heme; Hz: hemozoin) of MACS-enriched NF54*attB*^[ATeam1.03YEMK]^ trophozoite iRBCs after compound incubation for 6 h (CQ and 1o: multiples of ^72h^EC_50_; CHX: 50 µg/mL). Solitary 1o or CHX treatment (**A–F**) and CQ interaction (**G–L**) is presented as absolute amount of heme species, via absorbance of heme-pyridine complexes at A_410_ (**A–C and G–I**) and the relative fractions of the heme species distribution (**D–F and J–L**). (**A**) 100x ^72h^EC_50_ 1o reduces the Hb amount in the parasites (*P* = 0.0027). (**B**) 10x and 100x ^72h^EC_50_ 1o reduce the amount of “free” heme (10x: *P* = 0.0055; 100x: *P* < 0.0001). (**C**) CHX and 2x, 10x, 100x ^72h^EC_50_ 1o reduce the amount of Hz (CHX: *P* = 0.0004; 1o 2x: *P* < 0.0001; 10x: *P* < 0.0001; 100x: *P* < 0.0001). (**D**) 100x ^72h^EC_50_ 1o reduces the fraction of Hb (*P* = 0.016). (**E and F**) Solitary 1o or CHX incubations do not show effects on “free” heme or Hz fractions. (**G**) 100x ^72h^EC_50_ CQ treatment elevates the amount of Hb (*P* = 0.0004). Co-incubation with 2x, 10x, and 100x ^72h^EC_50_ 1o causes a reduced Hb amount compared to CQ treatment alone (2x: *P* = 0.0053; 10x: *P* < 0.0001; 100x: *P* < 0.0001). (**H**) 100x ^72h^EC_50_ CQ treatment elevates the amount of “free” heme (*P* = 0.0325). Co-incubation with 10x and 100x ^72h^EC_50_ 1o causes a reduced “free” heme amount (10x: *P* = 0.0005; 100x: *P* < 0.0001). (**I**) 100x ^72h^EC_50_ CQ treatment reduces the amount of Hz (*P* < 0.0001). Co-incubations with CHX or 1o do not alter the Hz amount. (**J**) 100x ^72h^EC_50_ CQ causes higher Hb fractions than vehicle control parasites (*P* < 0.0001). Co-incubation with 1o (2x: *P* = 0.005; 10x: *P* < 0.0001; 100x: *P* < 0.0001) causes reduced Hb fractions. (**K**) 100x ^72h^EC_50_ CQ causes higher “free” heme fractions than vehicle control parasites (*P* < 0.0001). Co-incubation with 1o (10x: *P* < 0.0001; 100x: *P* < 0.0001) causes reduced “free” heme fractions. (**L**) 100x ^72h^EC_50_ CQ causes lower Hz fractions than vehicle control parasites (*P* < 0.0001). Co-incubation with CHX (*P* = 0.0408) and 1o (2x: *P* = 0.0005; 10x: *P* < 0.0001; 100x: *P* < 0.0001) causes increased Hz fractions. *n* = 7 independent experiments derived from 1.5 × 10^7^ parasites/treatment group. Statistical analysis was conducted via two-way ANOVA and Dunnett’s multiple comparisons test. Reference group for multiple comparisons is indicated as black circles. Asterisks indicate significance level (*P < 0.05; **P < 0.01; ***P < 0.001; ****P < 0.0001).

As arylamino alcohols such as MQ and quinine have been shown to interfere with CQ-mediated alteration of Hb and heme species (7, 10, 18, 19), we analyzed 1o’s effects on absolute amounts and relative fractions of Hb, “free” heme, and hemozoin (Hz) in trophozoites treated with 1o. As we incubated a defined number of parasites per group, the pyridine-heme complex measured at A_410_ is equivalent to an absolute amount of the respective heme species per parasite, while the relative heme fractions reflect a heme species distribution that is independent of the parasite size. As in the previous experiments, we incubated parasites for 6 h in the presence of increasing doses of 1o (0x, 1x, 2x, 10x, 100x ^72h^EC_50_) alone (Fig. 7A through F), or in co-incubation with 100x ^72h^EC_50_ CQ (Fig. 7G through L). To exclude that the 1o effect is due to a non-specific negative effect on parasite metabolism or viability, we used the protein synthesis inhibitor cycloheximide (CHX) as a control in all experiments.

As previously reported, assays of this kind show high background noise, likely due to the low basal parasitic Hb content of less than 1%. Nevertheless, measurement of intraparasitic Hb without CQ co-incubation reveals a statistically significant lowered Hb amount (Fig. 7A) as well as relative Hb fraction (Fig. 7D) in the group incubated with 100x ^72h^EC_50_ 1o compared to the control, while we could neither detect any effects of lower doses, nor of CHX (Fig. 7A and D). We detected a reduced occurrence of “free” heme after 10x and 100x ^72h^EC_50_ 1o treatment (Fig. 7B), as well as less Hz after 2x, 10x, and 100x ^72h^EC_50_ 1o and CHX treatment. However, the observed lowered “free” heme and Hz quantities caused by 1o do not cause a significant change in their relative fractions. For the relative “free” heme and Hz fractions, we could not detect any effects of 1o or CHX alone, even at high concentrations of 1o (Fig. 7E and F).

We detected increased Hb, increased “free” heme, and decreased Hz species caused by CQ, both on the absolute and relative level (Fig. 7G through L), which is in line with previous literature (8, 9). Compared to CQ alone, co-incubation with 1o showed a dose-dependent reduction of the absolute Hb amounts and relative fractions, already noticeable at its’ 2x ^72h^EC_50_ (Fig. 7G and J). For “free” heme, co-incubation with 1o caused a decrease in both parameters with a dose-dependent trend (Fig. 7H and K), which was statistically significant at 10x and 100x ^72h^EC_50_ 1o. Co-incubation with 1o did not show an effect on absolute Hz (Fig. 7I). However, it showed a dose-dependent increase of the Hz fraction (Fig. 7L), significant at 2x, 10x, and 100x ^72h^EC_50_ 1o, which should be reflected in a consequential alteration of the Hb and “free” heme species. Additionally, CHX showed a significant attenuation of the Hz fraction after CQ treatment, but levels did not reach those of the vehicle control (Fig. 7L).

### 1o preserves FV morphology under CQ treatment

**Fig 8:**
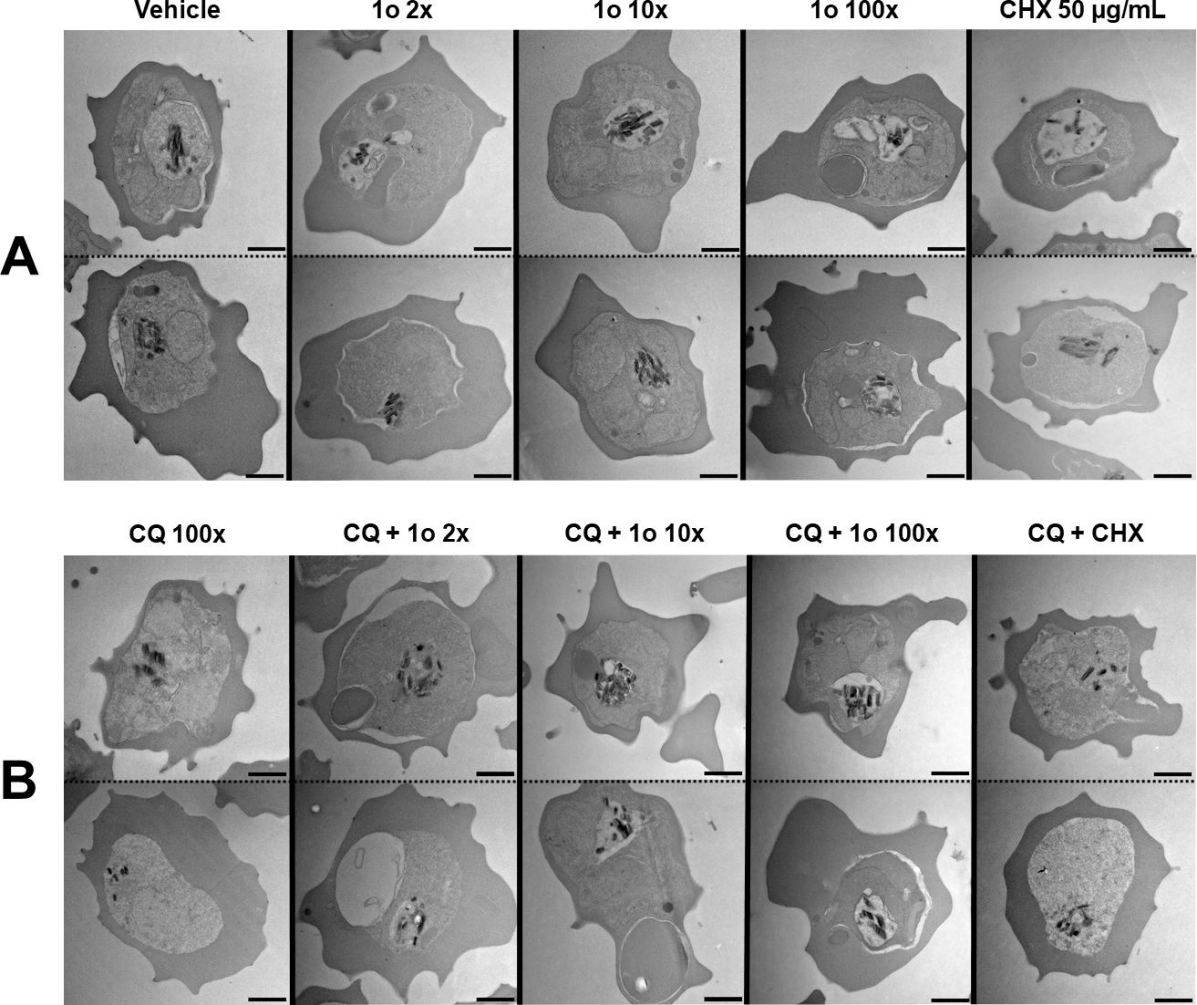
1o prevents CQ-mediated ultrastructural food vacuole alterations. TEM of MACS-enriched NF54*attB*^[ATeam1.03YEMK]^ trophozoite iRBCs after compound incubation for 6 h (CQ and 1o: multiples of ^72h^EC_50_; CHX: 50 µg/mL). (A) Vehicle-treated cells w/o co-incubation with 1o or CHX. (B) CQ-treated cells w/o co-incubation with 1o or CHX. Electron-dense hemozoin (Hz) is usually found within a distinguishable subcellular food vacuole compartment in vehicle-treated controls. Treatment with 100x CQ causes elevated parasite counts with disseminated Hz or Hz outside of a subcellular compartment. Co-incubation with 1o prevents such CQ-characteristic phenotypes. CHX does not appear to prevent the CQ action. Two representative images are shown. Scale bar equals 1 µm.

**Fig 9:**
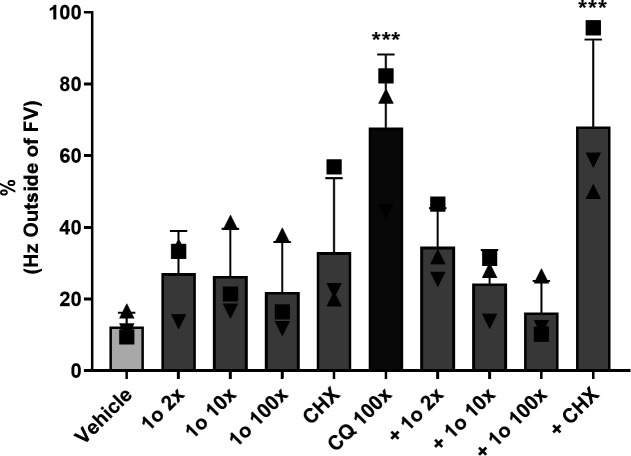
1o prevents CQ-mediated Hz dissemination. Quantification of TEM images of MACS-enriched NF54*attB*^[ATeam1.03YEMK]^ trophozoite iRBCs after compound incubation for 6 h (CQ and 1o: multiples of ^72h^EC_50_; CHX: 50 µg/mL). CQ (*P* = 0.003) and CQ + CHX (*P* = 0.003) treatment cause a significant increase in parasites with Hz outside of an FV structure compared to vehicle control. CHX alone and 1o alone or in co-incubation with CQ do not cause significantly increased levels. Readout is the percentage of parasites with Hz outside of a subcellular parasitic FV compartment of all parasite images with noticeable Hz crystals. *n* = 3 independent experiments with 12–97 Hz-containing parasite sections per data point. Statistical analysis was conducted via two-way ANOVA and Dunnett’s multiple comparisons test. Each group was compared to the mean of the vehicle control. Asterisks indicate significance level (***P < 0.001).

Drug treatment, including using CQ, is known to alter the ultrastructure of trophozoites, including the FV (17, 20–22). We used transmission electron microscopy to investigate if 1o also caused such alterations, and additionally, if 1o was able to prevent the changes observed upon CQ treatment. For quantification, we trained a "blinded" non-parasitologist to count the percentage of parasites with Hz outside of a distinguishable subcellular parasite compartment, equivalent to the FV, of all parasites with visible Hz crystals. Without treatment (Vehicle), we observed a spectrum of different phenotypes, ranging from Hz being contained within a clearly defined-membrane delineated compartment, representing the FV (Fig. 8A, Vehicle, upper panel) to strongly condensed Hz which was less clearly separated from the cytosol by a bounding membrane (Fig. 8A, Vehicle, lower panel). This variation is likely due to differences in parasite stage or as a result of sample preparation. Treatment with 1o alone did not appear to lead to any pronounced phenotypic changes, and a similar population of phenotypes was observed (Fig. 8A, 1o 2x − 100x). CHX appeared to cause a change in the phenotype population, with Hz now seen less condensed and free in the parasite cytosol (Fig. 8A, CHX 50 µg/mL). As previously reported, CQ caused Hz to be less condensed and not found in a clearly distinguishable subcellular parasite compartment (Fig. 8B, CQ 100x), and co-treatment with CHX led to similar results (Fig. 8B, CQ + CHX). Co-incubation with CQ and increasing concentrations of 1o shifted the population phenotype away from the CQ appearance, more toward the appearance of untreated parasites (Fig. 8B, CQ + 1o 2x − 100x). These observations are consistent with the statistical analysis of *n* = 3 independent TEM replicates, apart from that of CHX alone (Fig. 9).

### Late trophozoites are less affected by 1o than ring or early trophozoite stage parasites

**Fig 10:**
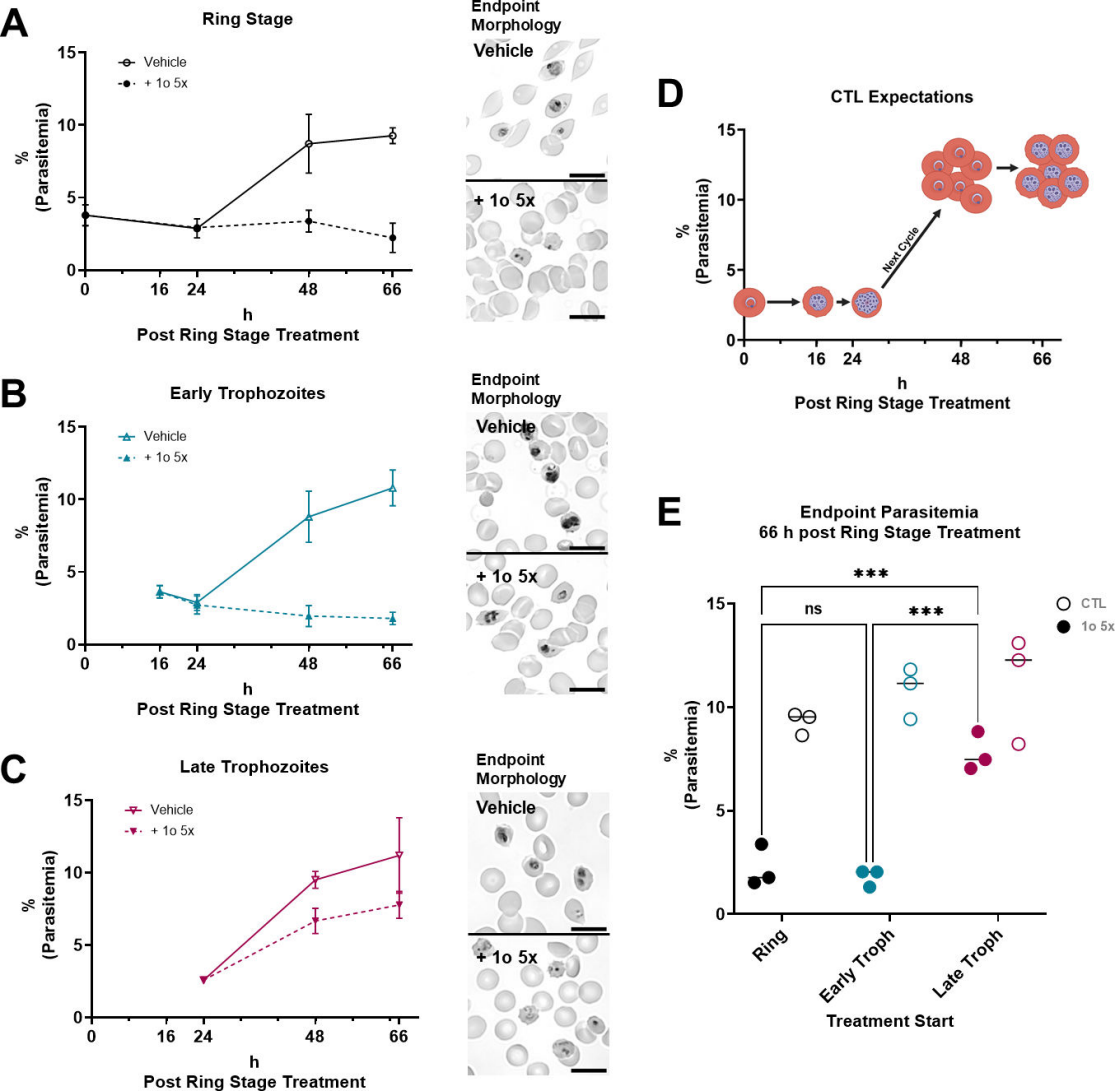
1o-treated late trophozoites can complete their life cycle until the following ring stage, in contrast to early trophozoites or ring stages. NF54*attB*^[ATeam1.03YEMK]^ ring stage (**A**), early trophozoite (**B**), and late trophozoite (**C**) iRBC cultures were treated with 5x ^72h^EC_50_ 1o (closed symbols, dotted line) or vehicle control (open symbols, solid line) and monitored for morphology and parasite count at the indicated time points. (**D**) Ring stages are expected to develop into trophozoites and then schizonts prior to merozoite egress, second cycle invasion, ring stage, and trophozoite development. (**A**) After 66 h, control parasites (Vehicle) proliferated and developed into trophozoites, while 1o-treated parasites did not increase parasitemia, nor develop into trophozoites. (**B**) 1o-treated early trophozoites on the same time scale did not proliferate and continuously appeared to be early trophozoites. (**C**) 1o-treatment of late trophozoites allowed proliferation into new ring stages, but not into trophozoites. (**E**) The endpoint parasitemia of each incubated parasite stage in the 1o-treatment and control groups was compared to each other’s parasite stage in each group. The parasitemia in late trophozoite 1o-treated cultures is significantly different from ring (*P* = 0.0007) and early trophozoite (*P* = 0.0004) 1o-treated cultures. Parasitemia of all vehicle-treated stages is not significantly different. Statistical analysis was conducted via two-way ANOVA and the Tukey multiple comparisons test. Asterisks indicate significance level (***P < 0.001). *n* = 3 independent experiments; error bars indicate standard deviation. Scale bar equals 10 µm. Created in BioRender.

To narrow down the targeted metabolic pathway of 1o’s relevant parasite-killing activity, we treated different parasite stages (ring stage, early trophozoite, late trophozoite) of infected red blood cell (iRBC) culture with a fixed dose of 1o (5x ^72h^EC_50_) or a vehicle control and monitored morphology and parasitemia over time (Fig. 10A through C). Untreated ring stages are expected to develop into trophozoites and then schizonts prior to egress as merozoites, second cycle invasion, ring stage, and trophozoite development (Fig. 10D). Ring stages were sampled just before and 24, 48, and 66 h post-treatment and used as a reference time scale for all other stages. When initiating treatment in ring stages, parasites did not, in contrast to the control group, propagate over the course of the experiment (Fig. 10A). Light microscopy revealed that, while control parasites progressed in their developmental stage to trophozoites, treated parasites took on a condensed morphology, likely representing degenerate ring stages (Fig. 10A). Treatment of early-trophozoite stage parasites in the same manner showed similar results, but with parasite growth in the treatment samples now blocked at the early trophozoite stage (Fig. 10B). Initiating the experiment with late-trophozoite stages, we observed that both treated and non-treated parasites increased in number over time (Fig. 10C). However, while untreated parasites, upon new invasion, developed into second generation trophozoite stages, parasites in the treated samples were blocked in their second cycle of development at the rings stage and again appeared degenerated (Fig. 10C). A comparison of all endpoint parasitemias from the above experiment revealed that the development of late trophozoites is significantly less compromised compared to treatment of ring and early trophozoite stages (Fig. 10E).

## DISCUSSION

We aimed to investigate the MoA of 1o and present here the first detailed study of its effects on parasites at the cellular level. We applied our established ATeam1.03YEMK ATP assay for our measurements in trophozoite stages (4, 11) to detect changes in cytosolic [ATP]. We ruled out confounding factors such as temperature, differences in parasite development, and pH through simultaneous measurement of 1o- and vehicle-treated cells within the same microscopic dish and control measurements with the pH sensor sfpHluorin. Strikingly, together with the parasite size, 1o’s response pattern at 10x ^72h^EC_50_ reveals parallels to MQ’s and LUM’s responses. Both cause an increased [ATP], a similar pH decrease, and reduced parasite size (4). An increase in cellular [ATP] can be caused by increased ATP production or decreased consumption. ATP is likely to be in high demand for protein synthesis in the metabolically highly active trophozoite stages, and this is backed up by our previous observations that inhibition of protein synthesis via CHX causes an increase in [ATP] (4). Therefore, one possible explanation for the observed effects could be inhibition (either direct or indirect) of protein synthesis, which could also result in reduced parasite size.

Following up on the parallels to MQ, we analyzed 1o’s interaction with CQ. CQ inhibits heme detoxification to Hz crystals (19), while it also inhibits Hb degradation (18), as well as heme detoxification via glutathione (14, 23). Ultimately, this leads to elevated levels of toxic free heme (9) and heme-inhibitor complexes (15), which are thought to kill the parasites (24), including lysis of the FV (17) and proteolysis in the cytosol (16, 17, 21). Drug effects can unfold on many levels and are dependent on incubation conditions, compound concentration, parasite strain and density, and incubation time. Within our assay conditions, with trophozoite iRBCs treated with 100x ^72h^EC_50_ CQ for 6 h, we could replicate phenotypes that are already described in the literature. These include a characteristic shift of ATeam1.03YEMK and sfpHluorin single-cell distribution, ATeam1.03YEMK and PfHSP90 degradation (4), Hb and “free” heme fraction increase, Hz fraction decrease (8, 9), and FV alterations with Hz dissemination (17). Taken together, our observations can be explained through CQ-mediated Hz formation inhibition, with concomitant free heme increase that could lyse the FV and cause proteolytic degradation in the cytosol. We use this concept for the interpretation of our interaction analyses.

We found that 1o prevents CQ-mediated ATeam1.03YEMK and sfpHluorin ratio drop and ATeam sensor degradation, similar to the effect noted for MQ (4). We found the interaction ^1o-CQ^EC_50_ to be in a similar range as the parasite-killing ^72h^EC_50_ of 1o. This suggests that the mechanism involved in the 1o block of CQ effects is indeed highly likely to represent the parasite-killing mechanism of 1o itself. In line with this, 1o prevents the ATeam1.03YEMK sensor degradation in a dose-dependent manner, which could reflect the bi-modal ATeam ratio drop. It should be emphasized that, under these conditions, the sensor readout does not represent changes in [ATP] but rather is being applied as a measure of sensor degradation.

Our 1o-CQ sensor interaction studies uncovered parallels to MQ and therefore implicate a similar interference with the parasites’ heme metabolism. To study this, we analyzed the relevant heme fractions in the parasites (9). Within our assay conditions, we can reproduce previous values for CQ-treated trophozoites (9), such as increased Hb, increased “free” heme, and decreased Hz fractions. For 1o treatment alone, we detected reduced absolute amounts of Hb, “free” heme, and Hz. While the relative “free” heme and Hz fractions did not significantly change under 1o treatment, these observations could be explained by general impaired parasite growth. Additionally, the level of "free" heme in the cytosol of blood stage parasites appears to be tightly regulated throughout its lifecycle and might possibly be balanced by import of heme via new permeability pathways (25, 26). Nevertheless, we could detect a decreased relative Hb fraction, which implicates altered Hb metabolism. While we could not detect a significant effect on the Hb fraction with concentrations lower than 100x ^72h^EC_50_ 1o, this may be explained by the low signal-to-noise ratio of the data produced under these conditions. Remarkably, within conditions of elevated Hb levels caused by CQ, 1o causes decreased Hb levels already at its 2x ^72h^EC_50_. Additionally, 1o also causes decreased “free” heme levels starting with its 10x ^72h^EC_50_ within the same scenario. At the same time, the absolute Hz amount is not affected by 1o. CHX, in contrast, does not affect the analyzed heme species in a similar manner, excluding a general effect caused by reduced parasite viability. Therefore, this data led us to conclude that the observed 1o effects and CQ interaction do not reflect unspecific effects caused by downstream inhibition of protein synthesis or simple parasite death, but rather support a mechanism for 1o interference in either uptake of Hb or Hb digestion to “free” heme. 1o has been reported to inhibit Hz formation *in vitro* (2). *In cellulo*, we could not detect an increase in the “free” heme fraction, suggesting that such effects are unlikely to represent the main parasite-killing activity of 1o.

We analyzed the ultrastructure of CQ- and 1o-treated iRBCs to investigate whether our biochemical findings match with the morphological changes of the parasites. CQ is known to cause the appearance of membranous structures within the FV, Hz in unusual vacuoles (20), as well as less discrete Hz crystals, vacuole rupture, and Hz outside of the FV (17). Additionally, using electron spectroscopic imaging, Combrinck et al. detected increased cytoplasmic iron in trophozoites after CQ treatment, indicative of heme diffusion outside of the FV and association with organelle membranes, which possibly kills the parasites (9). In line with this, we detected Hz dissemination into the cytosol, while 1o treatment already at 2x EC_50_ prevented the CQ effect. We detected increased “free” heme fractions caused by CQ under identical conditions, with 1o limiting this increase. A possible explanation for our observations could be that the CQ-mediated heme accumulation causes FV lysis with consequential Hz dissemination, which can be prevented by either directly or indirectly limiting heme accumulation.

Based on our hypothesis that 1o could interfere in a process within or upstream of Hb digestion, we analyzed its effect on the parasite development of different metabolic stages. We found that 1o-treatment blocked further development of ring or early trophozoite stage parasites, while parasites treated at the late trophozoite stage continued their lifecycle at a level approaching controls. This slight difference may be due to a small number of slightly asynchronous parasites in the population. This data suggests that a relevant metabolic pathway affected by 1o’s parasite-killing mechanism must be especially active during ring and early trophozoite development. The development from ring stage to early trophozoites is characterized by rapid growth and uptake of host Hb, while the subsequent late trophozoite and schizont stages are characterized by nuclear and eventual cellular division to merozoites (27). Together with our findings on the interaction of 1o with CQ, our data are consistent with a model in which 1o kills parasites by affecting their ability to uptake enough Hb to fulfil their metabolic requirements.

Our data give us a first idea of 1o’s MoA. We propose that 1o’s parasite-killing activity disrupts a metabolic pathway that is crucial for trophozoite development, possibly interfering with Hb uptake or digestion, and, thereby, superimposing CQ action. Our study is far from target identification, and 1o’s MoA may be pleiotropic in nature. However, we could narrow down a relevant metabolic pathway, which will benefit from the integration of further studies regarding resistance and omics analyses that are beyond the scope of our manuscript.

## MATERIALS AND METHODS

### Expression and purification of recombinant ATeam and sfpHluorin protein

ATeam10.3YEMK (11) and sfpHluorin (12) sequences in pQE-30 in *E. coli* strain M15 were used for overexpression and protein purification as previously published (4).

### Drug-sensor interaction studies

Drug-sensor interaction studies with ATeam1.03YEMK and sfpHluorin were conducted as reported (4). In brief, pre-heated ATeam1.03YEMK protein in ATeam buffer (100 mM HEPES, 50 mM KCl, 0.5 mM MgCl_2_, pH 7.34) with a final concentration of 1 µM was measured in black half-area 96-well plates in a Clariostar plate reader (BMG Labtech, Ortenberg, Germany) together with 1o (10 nM–1 µM), 10 mM MgATP^2−^ solution, or dimethyl sulfoxide (DMSO) vehicle control at 37°C after incubation for 5 min. Background-corrected emission ratios were calculated by division of 527/10 nm and 475/10 nm emission after excitation at 435/10 nm, respectively. Mean values were calculated from *n* = 3 independently produced proteins. For sfpHluorin, preparation was conducted accordingly. Measurements were conducted in sfpHluorin buffer (100 mM potassium phosphate, 100 mM NaCl, and 0.5 mM Na_2_-EDTA, pH 7.0), control buffer pH 5 (MES-KOH, 100 mM NaCl, and 5 mM EDTA), and control buffer pH 9 (100 mM Tris-HCl, 100 mM NaCl, and 5 mM EDTA). Excitation ratio was calculated from 390/15 nm and 482/16 nm excitation at 530/20 nm emission, respectively. Compound and vehicle treatments were normalized to the H_2_O control.

### *P. falciparum* cell culture

Transgenic sensor-expressing NF54*attB*^[ATeam1.03YEMK]^ and NF54*attB*^[sfpHluorin]^ parasite lines have been previously described (4). Blood stage parasites were cultured as previously described (28). Briefly, the parasites were cultured in complete media (CM) composed of RPMI 1640 medium with 2.1 mM L-glutamine, 25 mM HEPES supplemented with 0.5% Albumax, 9 mM glucose, 0.2 mM hypoxanthine, and 22 µg/mL gentamicin at 3.3% hematocrit and 37°C under 3% O_2_ and 3% CO_2_, respectively.

### Sorbitol synchronization

Parasites were synchronized using a 5% D-sorbitol solution (29) for 8 min at 37°C for at least three consecutive cycles prior to measurements. To exclude confounding effects from the sorbitol treatment, parasites were allowed to complete their lifecycle after sorbitol treatment, before measurements. At the latest, 72 h post-last sorbitol treatment, the population of iRBCs was defined as the trophozoite stage and used for measurements. Early trophozoites refer to parasites that are 6 h younger.

### *In vitro P. falciparum* drug susceptibility assay

The half maximal effective concentration of 1o against *P. falciparum* NF54*attB*^[ATeam1.03YEMK]^ after 72 h (^72h^EC_50_) was performed using a SYBR Green I-based fluorescence assay (30). A 10 mM 1o stock solution dissolved in DMSO was diluted in CM for serial double dilutions in black half-area 96-well plates (final assay concentrations: 160, 80, 40, 20, 10, 5, 2.5, 1.25, 0.625, and 0.3125 nM) with two technical replicates for each measurement. Wells were equipped with synchronized ring stage parasites to 1.5% hematocrit with 0.5% parasitemia. After incubation for 72 h under standard conditions, plates were wrapped in aluminum foil and stored frozen at −80°C overnight. The plates were thawed for 2 h at RT. Afterward, plates were equipped with 1× SYBR Green I in lysis buffer (20 mM Tris-HCl, 5 mM EDTA, 0.16% wt/vol saponin, and 1.6% vol/vol Triton X-100) and incubated in the dark for 1 h. Subsequently, the fluorescence at 494 nm excitation and 530 nm emission was measured in a Clariostar Plate Reader (BMG Labtech, Ortenberg, Germany). ^72h^EC_50_ determination was conducted via fitting a four-parametric variable slope using GraphPad Prism (version 10.4.1 for Windows, GraphPad Software, Boston, Massachusetts, USA, https://www.graphpad.com/).

### Trophozoite enrichment via MACS

Trophozoite iRBCs were enriched by magnetic separation as previously published (4).

### Drug and vehicle treatment

Magnetic activated cell sorting (MACS)-enriched trophozoite iRBC cultures were treated with 100× stock solutions in DMSO (1o) or H_2_O (CQ). Each treatment and vehicle control group was treated with all respective solvents of the individual experimental setup and a maximal DMSO concentration of 1% (vol/vol). Vehicle controls indicate groups that were treated with all solvents of the respective experiment. Treated cells were incubated under standard culture conditions.

### Measurements of sensor cell lines via live-cell microscopy

ATP and pH measurements in NF54*attB*^[ATeam1.03YEMK]^ and NF54*attB*^[sfpHluorin]^ were conducted as previously published (4). In brief, drug- and vehicle-incubated iRBCs were washed with Ringer’s solution (122.5 mM NaCl, 5.4 mM KCl, 1.2 mM CaCl_2_, 0.8 mM MgCl_2_, 11 mM D-glucose, 25 mM HEPES, and 1 mM NaH_2_PO_4_, pH 7.4) and allowed to settle on pre-heated ibidiTreat μ-Dish 35 mm Quad microscopy dishes (ibidi GmbH, Gräfelfing, Germany) at 37°C for 10 min. Images were taken with an Axio Observer.Z1/7 microscope (Zeiss, Oberkochen, Germany) with plan-apochromat 63×/1.40 oil immersion DIC M27 objective and Axiocam 506 camera. ATeam1.03YEMK measurements were conducted using Zeiss filter set 47 (EX BP 436/20, BS FT 455, EM BP 480/40) and 48 (EX BP 436/20, BS FT 455, EM BP 535/30) for CFP and CFP-YFP-FRET emission signal, respectively. Excitation was facilitated using the Colibri 7 LED module 430 nm (300 ms, 90% intensity). Background-corrected YFP signal at 535 nm divided by background-corrected CFP signal at 480 nm equals FRET ratio. For sfpHluorin, the Zeiss filter set 38 HE without excitation filter (BS FT 495 [HE], EM BP 525/50 [HE]) and Colibri 7 LED module 525 nm and 475 nm was used (each at 300 ms, 30%). Background-corrected GFP signal at 525 nm and excitation at 385 nm divided by background-corrected GFP signal at 525 nm and excitation at 475 nm equals the excitation ratio. Image analysis was conducted via ImageJ Fiji (31) 1.54f. Each measurement was gained using the mean sensor ratio of 200 iRBCs. *n* = 5 independent experiments were conducted. Each microscopic dish contained a control group as a simultaneously treated reference.

### Nigericin pH calibration

pH calibration via nigericin (32) of NF54*attB*^[sfpHluorin]^ was conducted as previously described (4).

### Western blot analysis

For western blot analysis, 2.5 × 10^6^ MACS-enriched and 6 h compound-treated trophozoite iRBCs were washed in phosphate-buffered saline (PBS [pH 7.3–7.5, 154.004 mM, 5.599 Na_2_HPO_4_, 1.058 KH_2_PO_4_]) containing 1× cOmplete Protease Inhibitor Cocktail Tablets (Roche Diagnostics GmbH, Mannheim, Germany) and lysed using M-PER buffer (ThermoFisher Scientific, Waltham, MA, USA) containing 1× cOmplete Protease Inhibitor Cocktail Tablets for 10 min. After centrifugation of cell debris, the supernatant was incubated at 95°C together with 4× sample buffer + dithiothreitol and separated using sodium dodecyl sulfate (SDS)-polyacrylamide gel electrophoresis according to Laemmli (33). Separated proteins were blotted on polyvinylidene difluoride membranes and stained with Ponceau S protein stain. Intensity of the Ponceau S staining was used for normalization of gel loading (34). After washing in TBST (Tris-buffered saline with 0.05% polysorbate 20), membranes were blocked in 5% TBST milk and probed using α-GFP (1:1,000; Roche Diagnostics GmbH, Mannheim, Germany) antibody in 5% milk in TBST, followed by secondary goat α-mouse IgG HRP antibodies (1:10,000 Dako, Agilent Technologies Deutschland GmbH, Waldbronn, Germany) in 5% milk in TBST. Probing of the PfHSP90 housekeeping control was conducted via α-PfHSP90 (1:500, a gift of Addmore Shonhai) antibody in 5% milk in TBST, followed by secondary goat α-rabbit IgG HRP antibody (1:2,000; Dako, Agilent Technologies Deutschland GmbH, Waldbronn, Germany). Band intensities were quantified using GelAnalyzer 19.1. (available at www.gelanalyzer.com by Istvan Lazar Jr., Ph.D., and Istvan Lazar Sr., Ph.D., CSc).

### 1o-chloroquine interaction

Dose-response analysis of 1o-chloroquine interaction (1o-CQ) of sensor readouts and WB signals was conducted via fitting of a four-parametric variable slope using GraphPad Prism (version 10.4.1 for Windows, GraphPad Software, Boston, Massachusetts, USA, https://www.graphpad.com/) for each of the *n* = 5 independent replicate series of each experimental setup. Measurements were set up as outlined above. For measurements of NF54*attB*^[ATeam1.03YEMK]^ and NF54*attB*^[sfpHluorin]^ sensor readouts, a CQ-treated contemporaneous control was used for normalization of the 25th percentile sensor ratio in each 4-well ibidiTreat μ-Dish 35 mm Quad (ibidi GmbH, Gräfelfing, Germany) microscopy dish. The 25th percentile of CQ alone- and vehicle-treated cells was set to 100% and 0%, respectively.

### Heme species distribution

The pyridine assay for determination of heme fractions was adapted from Combrinck et al. (9) to our trophozoite assay setup. In brief, 1.5 × 10^7^ MACS-enriched trophozoite RBCs were incubated with drugs or vehicle control under standard incubation conditions for 6 h. Afterward, cells were centrifuged at 2,100 × *g* and freed from host Hb using saponin lysis buffer (0.02% saponin in PBS) 2× for 10 min at 37°C. Cells were washed with saponin lysis buffer, subsequently with PBS, and stored in 100 µL PBS stocks at −80°C. For analysis, stocks were thawed, and 50 µL H_2_O was added prior to sonication for 5 min. A total of 50 µL HEPES buffer (0.2 M pH 7.5) and 50 µL H_2_O were added, followed by centrifugation at 16,000 × *g* for 20 min. The supernatant was used for Hb determination. The remaining pellet was resuspended in 50 µL H_2_O and 50 µL 4% SDS, followed by sonication for 10 min, vortex mixing, and incubation at 37°C for 30 min. Afterward, 50 µL HEPES buffer, 50 µL 0.3 M NaCl, and 50 µL 25% pyridine were added, followed by centrifugation for 20 min at 16,000 × *g*. The supernatant was used for the determination of the “free” heme fraction. The remaining pellet was mixed with 50 µL H_2_O and 50 µL 0.3 M NaOH and sonicated for 15 min. After incubation at 37°C for 30 min, 50 µL HEPES buffer, 50 µL 0.3 M HCl, 50 µL 25% pyridine, and 150 µL H_2_O were added. The solution corresponds to the Hz fraction. The supernatant designated for Hb analysis was mixed with 50 µL 4% SDS and sonicated for 5 min prior to incubation at 37°C for 30 min. Subsequently, 50 µL 0.3 M NaCl and 50 µL pyridine 25% (vol/vol) in HEPES buffer were added. The solution corresponds to the Hb fraction. The supernatant designated for “free” heme analysis was filled up to 400 µL with H_2_O and corresponds to the “free” heme fraction. The corresponding fractions were analyzed for absorbance of the heme-pyridine complex at 410 nm in a Mettler Toledo UV5 Bio spectrophotometer (Mettler Toledo GmbH, Giessen, Germany). Mean of *n* = 7 independent experiments, each calculated as the mean of 5 technical replicates, were used for fraction determination. The relative fraction (%) was calculated via division of the absorbance of each fraction by the sum of the absorbance of all fractions.

### Transmission electron microscopy

For TEM samples, MACS-enriched trophozoite iRBCs were incubated with 100× compound stock solutions and/or vehicle control (1% final DMSO concentration) for 6 h in complete media under standard incubation conditions. After harvest, cells were fixed with 500 µL TEM-fixing buffer (2% glutaraldehyde, 2% paraformaldehyde, 100 mM CaCo pH 7.4, [Electron Microscopy Sciences, Hatfield, PA 19440, USA]) at RT. For fixation, cells were shaken overnight at 4°C with consecutive storage at 4°C. Prior to sample preparation, fixed cells were washed three times with 500 µM PBS. Cell pellets were mixed with equal volumes of 3% agarose solution and hardened on ice. Cells in agarose were cut into cubes of about 1 mm length and soaked in PBS. After incubation with 1% osmium tetroxide for 1 h, cells were washed three times with H_2_O for 5 min. Afterward, cells were incubated with uranyl acetate at 4°C overnight. Uranyl acetate was washed off twice with H_2_O for 10 min. Dehydration was conducted with increasing acetone aqueous solutions (30%, 50%, 70%, 90%) and twice with 100% acetone for 10 min each. Afterward, cells were soaked with increasing SPURR (Electron Microscopy Sciences) in acetone solutions (25%, 50%, 75%) for 45 min each and 100% SPURR overnight at 4°C. The next day, cells were immersed in fresh SPURR for 4 h at RT, embedded in gelatin capsules (Electron Microscopy Sciences), and polymerized for 12 h at 70°C. After polymerization, the resin was washed and cut to 70 nm slices. Images were taken using the Joel JEM-1400 Flash.

### Stage-specific lifecycle effects

We synchronized for ring stages via sorbitol synchronization. Parasites were allowed to complete their lifecycle before treatment and were defined as ring stage culture 48 h after the last sorbitol treatment. After a further 16 and 24 h, the parasites were defined as early and late trophozoites, respectively. For quantification and analysis of parasite morphology, Giemsa-stained blood smears were sampled at the indicated time points. For each group and *n* = 3 independent replicates, at least 1,000 parasites were counted.

### Statistical analysis

Parasite experiments were set up in the fashion of a randomized block design, with each parasite culture of an independent experiment resembling an individual block. Statistical analysis was conducted using GraphPad Prism (version 10.4.1 for Windows, GraphPad Software, Boston, Massachusetts USA, www.graphpad.com).

### Generation of figures

Figures 1 (https://BioRender.com/t80p940), 2 (https://BioRender.com/z33s681 and https://BioRender.com/i96g461), and 10 (https://BioRender.com/f26j068) were assembled using BioRender.com.

## SUPPLEMENTAL MATERIAL

Supplemental material aac.00332-25-s0001.pdf Fig. S1 and S2.

ASM does not own the copyrights to Supplemental Material that may be linked to, or accessed through, an article. The authors have granted ASM a non-exclusive, world-wide license to publish the Supplemental Material files. Please contact the corresponding author directly for reuse.
