# Supplementary material for: Arylmethylamino steroid compound 1o interferes with Plasmodium falciparum’s hemoglobin metabolism
Source: Antimicrob Agents Chemother. 2025 Jun 23;69(8):e00332-25. doi: 10.1128/aac.00332-25 (PMC12327009; doi:10.1128/aac.00332-25)
Supplement: Supplemental material — Fig. S1 and S2. [file aac.00332-25-s0001.pdf]

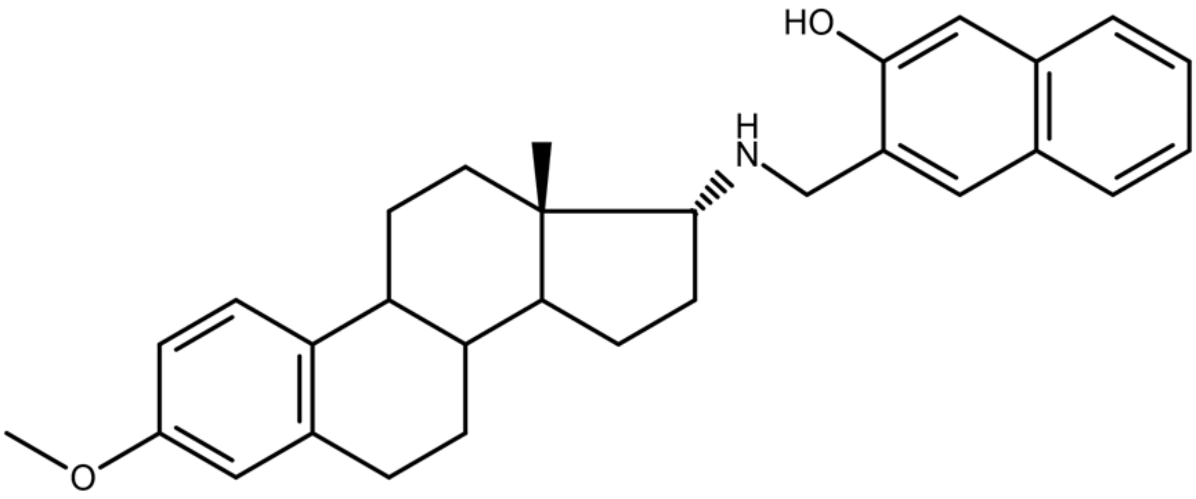

Fig. S1: Molecular structure of arylmethylamino steroid compound 1o

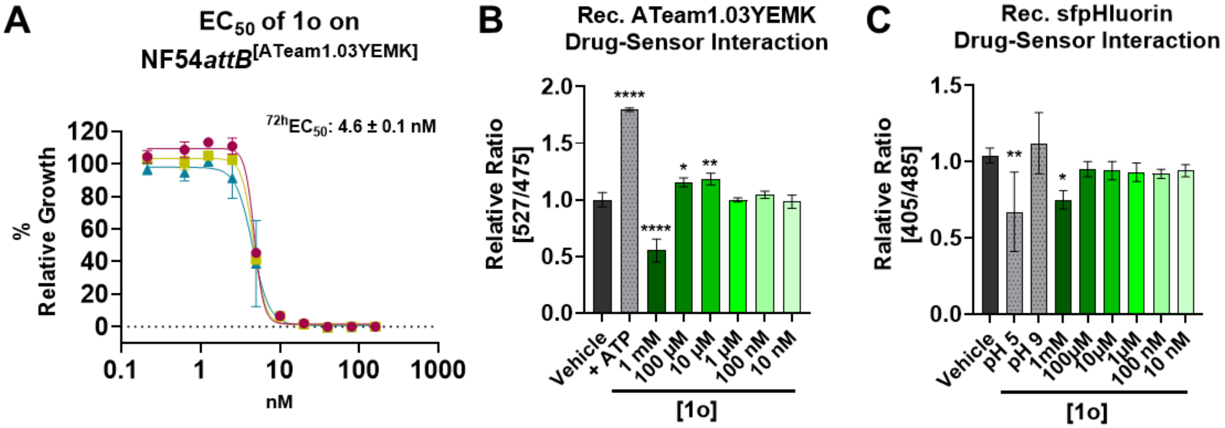

Fig. S2: 1o does not interfere with the ATP or pH sensor within biologically relevant doses. (A) Half maximal effective concentration on growth inhibition after 72 h ($^{72h}EC_{50}$) using SYBR green assay of 1o on the NF54*attB*[ATeam1.03YEMK] sensor cell line. Mean ± SD of n = 3 independent sigmoidal 4-parametric dose-response curves are shown. Each color (magenta (circles), yellow (squares), cyan (triangles)) represents an independent biological replicate. (B) Recombinant his-tagged ATeam1.03YEMK protein incubated with vehicle control, 10 mM ATP, or 1o (10 nM – 1 mM). Relative emission ratio of 527 nm and 475 nm after excitation at 435 nm in a plate reader. (C) Recombinant his-tagged sfpHluorin protein incubated with vehicle control, pH 5 and 9 buffer, or 1o (10 nM – 1 mM). Relative excitation ratio of 390 nm and 482 nm with emission sensing at 530 nm in a plate reader. n = 3 independent experiments are shown. Statistical analysis was conducted using ordinary one-way ANOVA and Dunnetts's multiple comparisons test. Error bars indicate standard deviation. Asterisks indicate significance level (*: $p < 0.05$; **: $p < 0.01$; ***: $p < 0.001$; ****: $p < 0.0001$) compared to the vehicle-treated control group.
